# Does the second opinion directive in Germany reach the patient? A parallel-convergent mixed-methods study

**DOI:** 10.1186/s12913-023-10197-0

**Published:** 2023-11-03

**Authors:** Susann May, Nadja Könsgen, Angelina Glatt, Dunja Bruch, Felix Muehlensiepen, Sonja Mählmann, Sebastian von Peter, Dawid Pieper, Edmund Neugebauer, Barbara Prediger

**Affiliations:** 1 Center for Health Services Research, Brandenburg Medical School Theodor Fontane, 15562 Rüdersdorf, Germany; 2 Institute for Research in Operative Medicine, Witten/Herdecke University, Witten, Germany; 3 Department of Cardiovascular Surgery, Brandenburg Heart Center, Brandenburg Medical School Theodor Fontane, 16321 Bernau bei Berlin, Germany; 4 Faculty of Health Sciences, Brandenburg Medical School Theodor Fontane, 16816 Neuruppin, Germany; 5 Institute for Health Services and Health System Research, Faculty for Health Sciences, Brandenburg Medical School, 15562 Rüdersdorf, Germany; 6 Brandenburg Medical School (Theodor Fontane), 16816 Neuruppin, Germany

**Keywords:** Second opinion, Surgery, Mixed-methods, Health system, patient-physician relationship

## Abstract

**Background:**

A Second Opinion Directive (SOD) was introduced in Germany in December 2018 for elective surgeries such as hysterectomy, tonsillotomy, tonsillectomy, and shoulder arthroscopy. The aim of the SOD is to avoid surgeries which are not medically induced and to support patients in their decision-making process. A physician who indicates an SOD-relevant procedure must inform the patient about the SOD and its specifications. At this time, it is not clear whether physicians provide information about the SOD to patients and whether and how the SOD is implemented in daily practice. Furthermore, nothing is known about how patients react when they are told that they have the right to seek a second opinion according to the SOD.

**Methods:**

To assess this, we undertook a parallel-convergent mixed-methods study with a qualitative and quantitative phase. Qualitative data were analysed by structured qualitative content analysis and survey data were analysed descriptively.

**Results:**

26 interviews were conducted with patients for whom one of the above-mentioned surgeries was indicated. In parallel, a questionnaire survey with 102 patients was conducted. The results show that the SOD is not implemented in Germany for the selected indications because patients were not informed as intended. At the same time, when the right to obtain a second opinion was explained, it seemed to have a positive effect on the physician-patient relationship from patients` perspective.

**Conclusions:**

It is possible that there is a lack of information for physicians, which in turn leads to an information deficit for patients. Better information for physicians might be part of the solution, but a negative attitude towards the SOD might also result in the low education rate. Therefore, in addition, potential patients or even the general population should be better informed about the possibility of obtaining a second opinion.

**Supplementary Information:**

The online version contains supplementary material available at 10.1186/s12913-023-10197-0.

## Background

For patients, seeking a second opinion (SO) is a way to get an additional opinion on a diagnosis, treatment, or prognosis from another independent physician. SOs are valuable when diagnostic accuracy is variable across physicians or access to high-quality care is restricted [1]. SOs can be used to strengthen the decision-making ability of patients [2, 3]. SOs can also help patients better understand their diagnoses, and consider diagnoses and treatment recommendations [4–6]. At the system level SO can also counteract overprescription of surgeries [7]. In Germany, there are various options for obtaining an SO. Statutory health insurers offer a wide variety of SO programs which are structured and subject to regulations. At the same time, patients in Germany have the right to freely choose their physician in the outpatient setting. It is quite common among patients to informally seek an SO with a physician chosen by the patient [8]. In this way, patients can see another physician for a second consultation to talk about their concerns without special authorisation. This practice is tolerated by the statutory health insurances and usually reimbursed without clear regulations. In other European countries, too, obtaining an SO is sometimes recommended or at least accepted. Whether this is paid for by the health insurance depends on the health care model of the respective country [9–11]. In December 2018, the Second Opinion Directive (SOD) was additionally introduced in Germany. Statutorily insured patients are entitled to a free independent SO if a hysterectomy, tonsillectomy, or tonsillotomy due to a non-malignant condition is indicated [12]. The directive that determines the design of the SO excludes malignant diseases for the SO. This is justified by the fact that the indications are more urgent and more often unavoidable. SO not according to the directive can be used in malignant diseases. In 2020, the indication of shoulder arthroscopy was added [13]. Other indications, such as the implantation of a knee endoprosthesis and amputation for diabetic foot syndrome, were also included in the SOD during the course of this project [14]. Patients can seek an SO from an authorised SO physician who has at least five years of full-time practice and a post-doctoral lecture qualification or is an advanced trainer for physicians. Each Association of Statutory Health Insurance Physicians provides an online list where authorised SO physicians can be found.

When providing one of the above-mentioned indications included in the SOD, the physician must also inform the patient at least ten days before surgery that an SO may be obtained. In addition, he or she must advise the patient where to find information about authorised SO physicians, that the physician or clinic who is going to perform the surgery may not provide the SO, that there is a decision aid, and that treatment records can be taken along. Moreover, physicians should provide a patient information leaflet. Afterwards, the patient can decide whether to seek an SO.

At this time, it remains unclear whether and how the SOD is implemented in the treatment of patients facing a decision to undergo an elective surgery. It is uncertain whether physicians provide information about the SOD. Furthermore, nothing is known about how patients react to being told that they have the right to obtain an SO, and what impact this has on the patient-physician relationship. Therefore, we investigated by means of a mixed methods study how information about the right to obtain an SO is currently provided and experienced from the patients’ perspective in order to address the following research questions:

- How is the SOD implemented for patients who are facing the decision to undergo an elective surgery?
- How is the information on the right to obtain an SO disseminated? How do physicians provide information on the procedural specifics of the SOD?
- How do patients react to the information that they have the right to obtain an SO? What impact does this have on the patient-physician relationship?

**Fig. 1:**
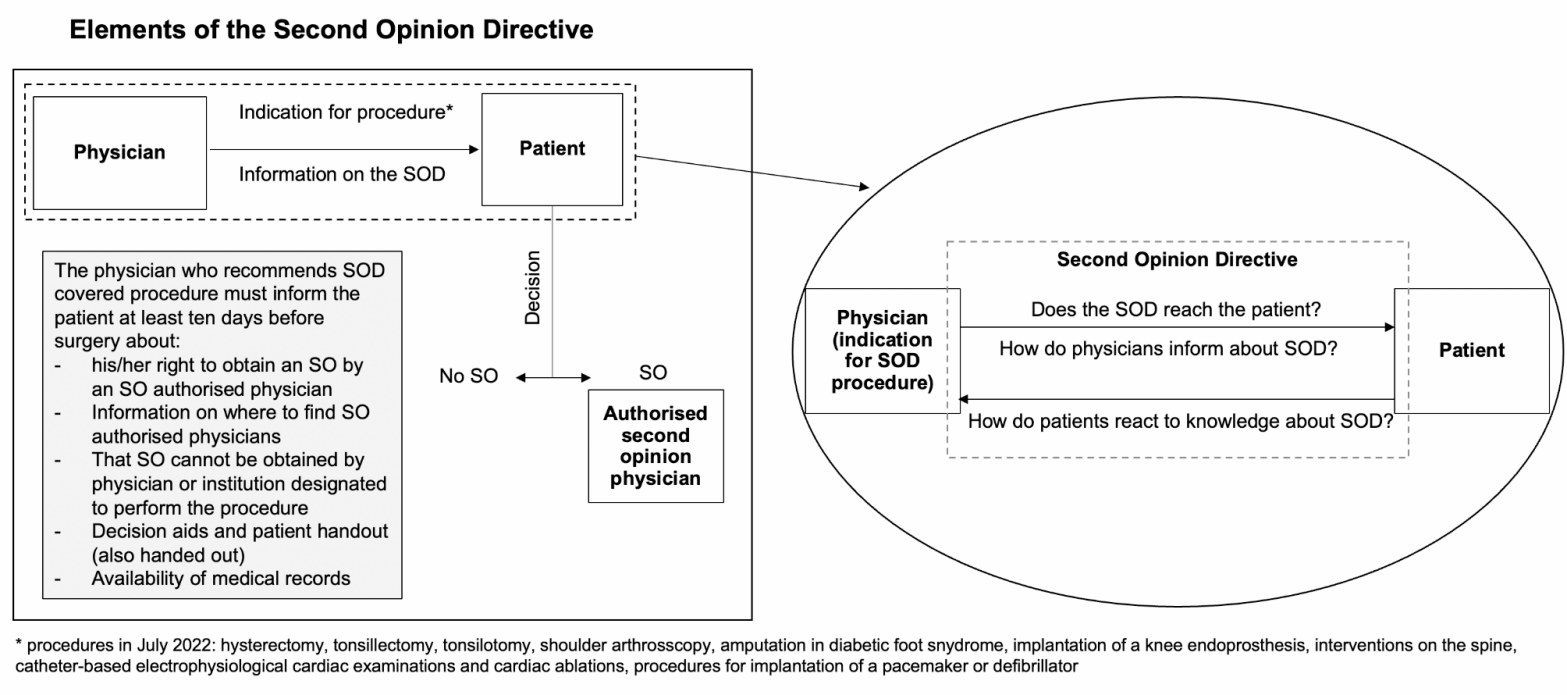
Depiction of the addressed elements of the SOD investigated in this study; SO = second opinion; No SO = no second opinion; SOD = Second opinion directive

The present study is part of the ZWEIT project [8], which examines the characteristics and use of SO programs in Germany and considers the resulting needs and wishes from the perspectives of physicians [15, 16] and of (potential) patients [17, 18]. Figure 1 combines the elements of the SOD investigated in this study and the research questions.

## Methods

### Study design

**Fig. 2:**
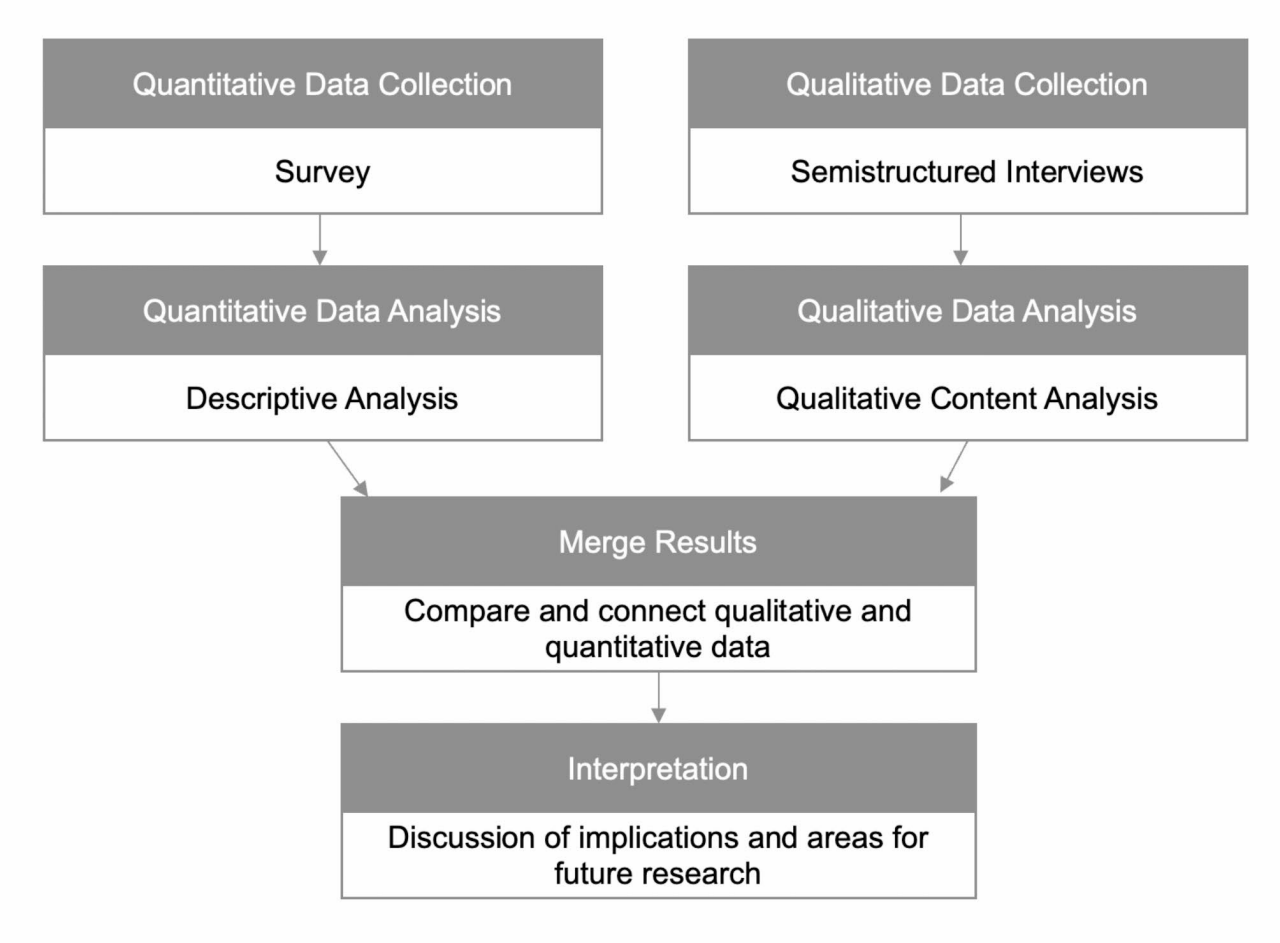
Study design

Because the implementation of the SOD is a complex procedure, and in order to gain a comprehensive and in-depth overview of this topic narratively and numerically, a convergent parallel mixed-method design was implemented. This parallel-convergent mixed-methods study was conducted in a quantitative and a qualitative phase. In this study, the qualitative and quantitative data was collected and analysed simultaneously and independently [19]. Data analysis was performed separately and merging was used for integration [20] (Fig. 2).

Participants in the qualitative research provided written consent. By returning the completed questionnaire, participants indirectly provided their consent to the quantitative survey. This study was approved by the Ethics Committee of the Brandenburg Medical School Theodor Fontane, Reference ID: E-01-20190529. This manuscript and its reporting align with checklists for the Consolidated Criteria for Reporting Qualitative Research (COREQ) [21] (Supplementary Material File 1) and the reporting of surveys [22] and the reporting of a mixed-methods study (GRAMMS) [23] (Supplementary Material File 2). The study group consisted of 8 members, 4 in the qualitative approach and 4 in the quantitative approach.

### Study population and recruitment

Participants were selected using purposive sampling, specifically using criteria aimed at including patients for whom tonsillectomy, tonsillotomy, hysterectomy, or shoulder arthroscopy was indicated. Further inclusion criteria were: insured by statutory health insurance, sufficient knowledge of the German language, and age ≥ 18 or parent or legal guardian, respectively, willing to complete the questionnaire or participate in the interview.

Initially, we recruited physicians specialised in otolaryngology, gynaecology and orthopaedics in Germany, based on registries from the Association of Statutory Health Insurance Physicians. We focused on outpatient settings because these physicians commonly provide the surgical indication for the above-mentioned procedures. Subsequently, the physicians recruited participants by distributing the questionnaires and invitations for telephone interviews to their patients or parents of their patients who met the inclusion criteria. Recruitment was scheduled to occur between 09/019 and 07/2021, but was extended to 01/2022 due to slow recruitment (Covid 19). Physicians from the states of Berlin, Brandenburg and Lower Saxony recruited patients.

Participating physicians received a remuneration of €5 for each invited patient (regardless of whether the patient participates or not). Eligibility for the qualitative part was verified prior to the interview as part of the scheduling of the interview by telephone, e.g., by asking, whether there was an underlying non-malignant disease. The interviewed participants received €40 for their participation. Participants of the survey received a 10 Euro gift card and the referring physician received €20 in cash or as a gift card. Participants could participate in both the interview and the survey. If a person participated in both parts, an allocation from the interview to the survey was not possible.

### Survey

Four researchers (AG, DP, BP, NK) developed three questionnaires consisting of closed questions only, one for patients with hysterectomy, one for patients with tonsillectomy/tonsillotomy, and one for the legal guardian of patients with tonsillectomy/tonsillotomy under the age of 18 years. We piloted the questionnaire with a sample of 25 patients from the university clinic Cologne Holweide. The questionnaire contained 35 questions divided into 6 parts: the conversation with the physician, the decision-making process regarding the surgery, the desire for an SO, demands and wishes regarding the SO physician and the SO procedure, experiences with an SO, and health-related and general questions (including sociodemographic data). Health literacy was assessed using the 16-item European Health Literacy Survey (HLS) [24]. The answer category ‘do not know’ was added to the 4 answer options (very easy, fairly easy, fairly difficult, and very difficult). We used the German version of the Decisional Conflict Scale (DCS) [25]. We excluded the effective decision subscale as the queried person had not made a decision at the time of the survey. The questionnaire for participants with an indication for shoulder arthroscopy was introduced in 12/2020 because the indication was later included in the SOD. We used only a short form with 11 selected questions from the original questionnaire as we hoped to receive more responses by doing so. The completed questionnaires were sent anonymously to Witten/Herdecke University, postage paid by receiver. We used the postal codes and the categorization of “degree of urbanisation” (DEGURBA) by Eurostat [26] and the regional statistics of the German Federal Statistical Office [27] to allocate data to settlement patterns. One person extracted the questionnaire data into an Excel spreadsheet developed a priori, and another person checked the accuracy of all extracted data. We used Microsoft Excel to analyse the results descriptively. We reported means, medians, and interquartile ranges (IQR).

### Qualitative interviews

To explore experiences regarding the information process for seeking an SO, semi-structured interviews [28] were conducted. The preliminary interview guide was drafted newly in a team including representatives of the disciplines of health sciences, psychology, and medicine (SMä, SMa, DB, SvP), with further help from clinical experts (physician-specialists in gynaecology, otorhinolaryngology, and orthopaedics). The interview guide was piloted in three interviews. Afterwards, minor editorial adjustments to the interview guide were necessary. The main topic areas explored were providing information regarding the right to obtain an SO, contents of the information provided by the treating physician, the impact of the SOD on the patient–physician relationship, and, if relevant, the procedure of obtaining an SO, see Supplementary Material File 3. To reduce infection risk due to the COVID-19 pandemic, the qualitative interviews were conducted via phone from March 2020 to December 2022 by SMä and SMa. The interviews were conducted either before surgery or a maximum of 3 weeks after surgery. In addition, socio-demographic data were collected, including gender, age, educational level, and job position of the interviewees. The interviews were audio-recorded and transcribed verbatim. Qualitative analyses were performed iteratively by two health researchers (SMä, SMa), based on Kuckartz’s structured qualitative content analysis [29] using MAXQDA software (Verbi GmbH). Inductive content analysis was used. First, the subject’s quotes were extracted and condensed into codes. Main categories and subcategories were formed from the codes. Consensus discussions were held continuously in the research group until a common understanding of all the emerging categories was achieved. The application of the category system was validated again by an internal review to ensure traceability, whereby two researchers independently applied the developed category system to the entire data (SMä, SMa). Data collection and analysis were circular and continued until no substantially new findings emerged and theoretical saturation was reached.

### Mixed methods analysis

Finally, we compared the quantitative data obtained from the questionnaires with the categories derived from the inductive qualitative analysis (i.e. the interviews). Data integration was performed by bringing the data together in a joint display, merging them in a visual way to provide new insights beyond the information gained from the separate quantitative and qualitative results [20].

The qualitative and quantitative data were combined at two stages of the study: At the design phase, a plan for collecting both forms of data was developed in a way that will be conducive to merging the databases. Similar contents were asked in both instruments, in the quantitative survey to analyze the frequencies, in the qualitative survey to assess the perception and the experience of the participants. Furthermore, the merging occurred after the statistical analysis of the numerical data and qualitative analysis of the textual data [20].

## Results

From 09/2019 to 01/2022, 69 physicians took part in the recruitment, 28 were specialised in gynaecology, 21 in otolaryngology, and 20 in orthopaedics.

### Survey

We received 102 completed questionnaires. Mean age was 44 years (IQR 19–59). In the groups hysterectomy and tonsillectomy/tonsillotomy, females constituted 84.5% (49/58) of the respondents. In the group tonsillectomy/tonsillotomy, 19 parents or legal guardians replied to the questionnaire, the median age of the children was 8 (IQR 6–11), and females constituted 47.4% (9/19) of respondents. In the groups hysterectomy and tonsillectomy/tonsillotomy, HLS was 14 in median (IQR 11–15), and the DCS resulted in 12.5 (IQR 4.2–31.3). Those three questions were not part of the short form for shoulder arthroscopy. Regarding professional education, 54.9% (56/102) reported having an apprenticeship, 30.4% reported having a university degree (31/102), and 14.7% (15/102) reported no professional education, were still in education, or did not give a valid response. The degree of urbanization was for 25.5% (26/102) high, for 36.3% (37/102) middle, for 27.5% (28/102) low, and 10.8% (11/102) could not be categorised. We asked ”Do you want a second medical opinion in your current situation” and 29.4% (30/102) replied with yes or rather yes and 69.6% (71/102) replied with no and rather no; there was one invalid answer. The characteristics are displayed in detail in Supplementary Material File 4.

**Fig. 3:**
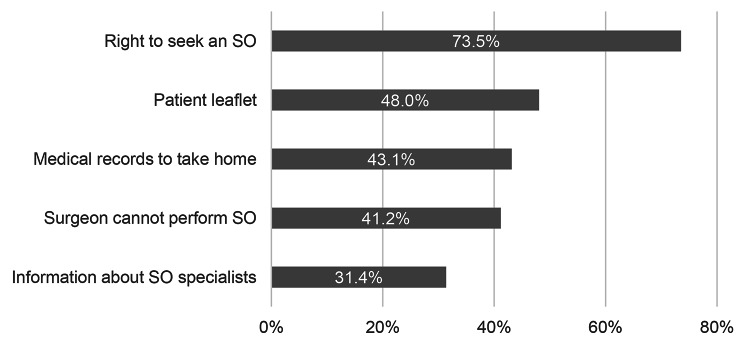
The patient was informed about…(N = 102)

We asked all participants if they were informed about specific aspects of the SOD, they replied “yes” to the following: the right to seek an SO 73.5% (75/102), the patient leaflet 48.0% (49/102), that they can take their medical records 43.1% (44/102), the prohibition of treatment by SO physicians 41.2% (42/102), and information where to find SO physicians 31.4% (32/102). Multiple answers were possible. See Fig. 3.

**Fig. 4:**
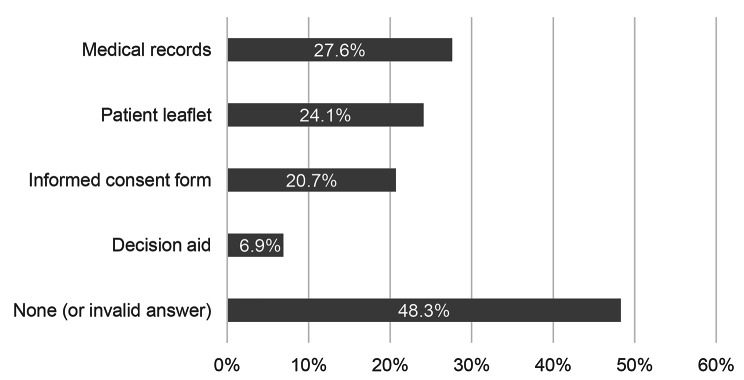
The patient received…(N = 58)

We asked participants with hysterectomy and tonsillectomy/tonsillotomy about the documents they received from their physician. Figure 4 shows their replies: 27.6% (16/58) received their medical records, 24.1% (14/58) received the patient leaflet, 20.7% (12/58) received the informed consent form and 6.9% (4/58) received the decision aid. 48.3% (28/58) did not (validly) reply to any of the items. Multiple answers were possible.

### Interviews

A total of 26 patients were interviewed. 6 participants had an indication for HE, 9 participants had an indication for TE or TT, and 11 participants had an indication for SA. The mean age of the participants was 48 years. The characteristics of the interviewees are shown in Supplementary Material File 5. The interviews lasted between 28 and 45 min (mean 35). In total, 13 patients sought an SO. However, these were informal SOs and not SOs in the sense of the directive.

As in the survey, not all patients were informed about the right to seek an SO by the physician providing the indication (although obligatory). In summary, 15 patients (57%) had been verbally informed that they have the right to obtain an SO and 6 patients (10%) had received the leaflet. The qualitative data gave insight into how information about the right to obtain an SO is actually provided. The corresponding coding tree is presented in Supplementary Material File 6.

Physicians inform patients that they can obtain an SO, but not in accordance with the SOD. The information on the procedural specifics of SOD does not reach the patients.

*“The physician said: it seems to necessitate a surgery. However, I am not a shoulder specialist. I am an orthopedist, but not a specialist on shoulders. I would like to refer you to a colleague for a second opinion.“* (114_SA, Pos. 4).

Furthermore, in some cases it is not the physician but the medical assistant who explains the right to obtain an SO.

*“It was the nurses who informed me and provided the information sheets.“* (113_SA, Pos. 33–36).

The information about the right to obtain an SO is sometimes not provided by the physician who gave the indication, but rather by the surgeon during the pre-surgery consultation.

*“I: Have you been informed that you have the right to obtain a second opinion?*

*P: He did not say it, but I know it.*

*I: and how did you get to know about this study?*

*P: Through doctor X. (surgeon).“* (107_HE, Pos. 27–30).

In some cases, there was a negative influence by the physicians during the explanation.

*„So, according to the feeling as he put it, it rather seemed as if he would find it idiotic that he needs to inform the patient about it now.“* (121_TT, Pos. 64).

If patients are given the patient leaflet, they usually skim over it and cannot reproduce its contents.

*„Perhaps I skimmed through it. Well, not consciously. I did not actually sit down and read it. Had I done so I could tell you more about it.“* (116_TT, Pos. 80–81).

Some patients reported that they do not remember receiving a patient leaflet.

*„No, I can’t remember [patient leaflet]. Maybe I did already close up then. It could be (laughs) that I already stopped listening.“* (119_SA, Pos. 33–34).

Physicians do not provide any further information on SO physicians.

*„Well, he didn’t tell me any names or further information on where I could find some. He said that I could try to find something myself.“* (120_SA, Pos. 16).

Physicians recommend obtaining an SO and give patients a referral to another physician. However, this is not a certified SO physician and this is not the purpose of the SOD.

*„The doctor immediately gave me the prescription and said: “I can recommend you this doctor. He is really good with shoulders.“. And then wrote out the prescription for me. But this wasn’t really a second opinion physician.“* (114_SA, Pos. 28).

In some cases, the right to seek an SO was mentioned after surgery.

*„Well, informing me (about the right to a second opinion) had been a bit late then, am I right […]. That was only after the surgery, when I was there for the follow up.“* (117_HE, Pos. 56–59).

Sometimes the right to seek an SO was explained one day before the surgery.

*“Exactly, it was at the pre operation discussion [getting informed about the second opinion]. I had a pre operation discussion, that has been it. One day before the surgery.“* (112_SA, Pos. 60).

None of the interviewees reported that they were informed that the SO cannot be provided by physicians or institutions designated to perform the procedure or that they can provide their medical records to the authorised SO physician. Nor were they informed about where to find decision aids. Table 1 summarises the expected process in the course of the information about the right to obtain an SO according to the SOD as well as variations that were observed in daily health care practice.

**Table 1:** Receiving information about the right to seek an SO from patients’ perspective

Element of information in the SOD	Expected Process	Variations of the process in daily health care practice
Informing about the right to obtain an SO	Physician verbally informs the patient that they have the right to obtain an SO.	Physicians inform patients that they can obtain an SO, but not in accordance with the SOD. The information on the procedural specifics of SOD does not reach the patients. Sometimes the medical assistant explains the right to obtain an SO or the surgeon. In some cases, there was a negative influence by the physicians during the explanation.
Patient leaflet	Physician hands out a patient information sheet for the patient.	Some patients reported that they do not remember receiving a patient leaflet or cannot reproduce its contents.
Information about authorised SO physicians	Physician provides information on available authorised SO physicians	Physicians did not provide any further information on authorised SO physicians or referred patients to physicians who were not authorised SO physicians.
10-day limit	Physician must inform the patient about the right to seek an SO at least ten days before planned surgery.	In some cases, the right to seek an SO was mentioned after surgery or directly before the surgery.
Surgery does not take place where the SO is given	Physician must inform the patient that the SO cannot be provided by physician or institution designated to perform the procedure.	Not reported.
Availability of medical records	Physician must inform the patient about the availability of medical records for the authorised SO physician.	Not reported.
Information about decision aids	Physician must inform the patient about decision aids.	Not reported.

### Patients’ experiences with being informed about the right to obtain an SO

In total 15 patients had been informed about the right to obtain an SO. The information about the right to obtain an SO is predominantly experienced positively and was appreciated by all patients. It was not experienced as uncertainty in the decision-making process. The characteristics of the experience are illustrated in the following section.

The information about the right to obtain an SO was experienced as a confidence-building activity. Participants felt the information increased their confidence in the physician and made them feel well treated.

*„Effects in the sense that I found it positive that she made me aware about the opportunity to obtain a second opinion and yes, which actually improved the personal trust. She could have also kept quiet about it and I would have read about it somewhere else then.“* (104_HE, Pos. 75).

In addition, informing the patient about the right to obtain an SO demonstrates the physician’s competence in the sense that he or she is confident about the medical treatment.

*„Well, I mean, sometimes the doctor exposes himself. When he says: “You can obtain a second opinion”. This basically says: I point it out to you, inquire about it and come back. He is sure about it. It shows competence.“* (112_SA, Pos. 160).

Patients perceived the information about the right to obtain an SO as a reassurance of the diagnosis. According to this, the information supports quality of care and gives patients confidence in their decisions.

*„I think, in principle, I would consider it good if my doctor would tell me in such a manner, because it gives you the feeling that one can assure oneself with someone else. So, he doesn’t try to fiddle or something.“* (117_HE, Pos. 117).

In addition, informing patients of their right to obtain an SO also encourages them to think about their disease and how they manage it. Consequently, the information leads, among other things, to critical reflection and engagement with health-related information.

*„Yes, maybe that more people will use it and perhaps think about it even more whether this all is actually right, what the doctor was saying for instance. Yes. Maybe some surgeries could be, yeah, maybe sometimes one would not need to operate that quickly then as it is the case. Maybe there would be other possibilities to help the patient and not to operate. Also, a narcosis always entails a certain risk.“* (102_TE, Pos. 105).

### Mixed-methods-findings

The comparison of the quantitative and qualitative data generated six confirmed findings whereby the qualitative data permitted a deeper understanding of the implementation of the SOD. The Mixed-Methods interpretation approaches are presented in Table 2.

**Table 2:** Mixed-methods interpretation of the qualitative and quantitative findings

Topic	Mixed-methods interpretation	
Information about the right to seek an SO	Results permit a deeper understanding of how education is implemented in care. Patients were not informed as intended by the directive, in terms of both frequency and content. Possible insecurity of the patient due to negative attitude of the physician. The SOD does not arrive in care.	
Patient information leaflet handed out	Low numbers of those who reported receiving the leaflet are supported by the qualitative data. Some do not remember the content, which may indicate low information content or irrelevance to the patient. It might be useful to examine the usability of the leaflet in other surveys.	
Surgery does not take place where the SO is given (prohibition of treatment by the SO physician)	Low numbers of those who reported being informed about the prohibition of treatment by the SO physician. The fact that nobody mentioned this aspect in the interviews supports the quantitative data.	Possibly the physicians did not have enough knowledge about the detailed contents and specifications of the SOD.
Availability of medical records	The information that medical records can be taken along does not reach the patients sufficiently. The fact that nobody mentioned this aspect in the interviews supports the quantitative data.	
Received decision aid (physician only needs to inform about)	In individual cases, the decision aid was handed out. The fact, that nobody mentioned this aspect in the interviews supports the quantitative data.	
Information about authorised SO physician	Low numbers of those who reported being informed about where to find an SO physician are supported by the qualitative data. The information where to find SOD physicians does not reach the patients.	

*Question only in hysterectomy, tonsillectomy/tonsillotomy questionnaire

## Discussion

This parallel-convergent mixed-methods study explored the current implementation of the German SOD, which aims to reduce the number of potentially unnecessary surgeries and to improve medical decision making from patients` perspective. As far as we know, this is the first study which has addressed the information about the right to obtain an SO in the context of the SOD and its specifications. The results of our interviews and survey indicate that the SOD is not yet implemented in Germany for the selected indications. At the same time, when the right to obtain an SO is explained, it seems to have a positive effect on the physician-patient relationship. With the help of quantitative and qualitative data sources, we were able to identify challenges that occur during implementation. Based on our data, we have formulated approaches to address these challenges in the following.

Other studies that examined SO seeking in general showed that there are factors that promote SO seeking. A systematic review by Greenfield et al. showed that those who seek an SO are rather female, middle aged and have a higher socioeconomic status [30]. In our quantitative study we did not analyse data on correlation as we only had 30 persons who replied to “Do you want a second medical opinion in your current situation” with “(rather) yes”. However, we saw in the group of Tonsilltomy/Tonsillectomy that those who replied with “(rather) yes” (n = 9) were female except one person. We didn’t ask about gender in the other groups. In one other study we saw, that the chosen indications for the SOD were not the most relevant for patients, which were disc surgery and joint replacement, prostatectomy and meniscus resection [31]. We are not aware of any work studying the dissemination of an SOD like we did, but there are at least similar questions regarding the information about SOs in general. Groß et al. found out, that physicians informed 35.7% of patients with breast cancer about the possibility of seeking an SO. The probability of doing so was higher, when the patient had a higher education and was younger than 75 years old [32].

The main finding is that the SOD does not reach the health care system because physicians do not provide information as intended in the SOD. While physicians are generally positive about the concept of SOs, a previous study demonstrated that the implementation of SOD is associated with a lack of acceptance and challenges in everyday practice [15]. Insufficient implementation could be explained, on the one hand, by the fact that physicians tend to have a negative attitude towards the SOD. However, it remains unclear whether a lack of knowledge about the SOD could have led to not all aspects being explained. Since the SOD came into force, there have been five amendments to the directive. Perhaps the complexity of the directive coupled with a lack of interest is a reason why many physicians are not fully informed. However, there may be a negative influence on patients when physicians reveal their negative attitude towards the SOD. Patients are confronted with the challenge of dealing with this potentially conflictual situation. Patients may be worried regarding the decision for or against an SO but also about the relationship to their physician.

In particular, the fact that physicians do not provide information on where patients can seek and find authorised SO physicians can be seen as challenging in the context of implementation. One of the reasons for the introduction of the SOD was to relieve the patient of the need to search for a suitable SO physician. According to the SOD, the physician who indicates the procedure must inform the patient which website to use to find an authorised SO physician. The patient may search there and select a SO physician; digital literacy is assumed. If the physician does not provide information where to find the SO physician, the patient is on his/her own to find an authorised SO physician. The patient could do some research on his or her own or ask his or her insurance company about an SO physician. But in most cases, the only option is to seek an informal SO from a physician not authorised by the SOD. To find information on the therapy of a disease, where to find professional help, and to estimate if an SO is needed are abilities associated with sufficient health literacy [33]. A certain level of health literacy seems to be a requirement to find a suitable physician for this informal SO. Some physicians recommend other physicians for an SO, but these are then neither authorised by the SOD, nor is independence guaranteed. Moreover, it is unclear whether patients have access to authorised SO physicians to obtain an SO. A first study indicates that the spatial distribution of SO physicians in Germany varies from region to region [16]. Further research is needed to determine if obtaining an SO from an authorised SO physician is feasible and also if the quality of an SO from an authorised physician is higher than from a nonauthorised physician. The relationship between the qualification criteria for authorisation as an SO physician and the quality of the SO has not yet been studied.

This is also related to the education about the prohibition of further treatment. The physician must inform the patient that the SO is not allowed to be obtained where the surgery is carried out. Again our study showed low numbers of education on that item of the SOD. However, it is necessary that the patient knows that no financial interests should influence the provision of the SO. In another survey, we found that patients sometimes interpreted the pre-operation discussion as an SO (data not yet published). This shows how important a common understanding of SOs is and that independence of the SO physician is part of it.

The indicating physician also needs to inform about and provide the patient leaflet. We saw few participants reporting that they received the leaflet. Here we can raise more questions than answers: Is the patient information leaflet a meaningful and useful way of informing patients in the decision-making process? Patient understanding of health conditions and treatment options is critical to a successful physician-patient dialogue. However, the availability of patient information leaflets does not guarantee access to quality information tailored to the needs of each patient [34]. A study on patient information pamphlets in urogynaecology has already demonstrated that the comprehensibility and effectiveness of patient information pamphlets is determined by various aspects such as layout, illustrations, and readability [35]. To assess the usefulness and effect of the patient information leaflets on patients’ decision-making process, further research is needed.

Additionally, according to the SOD, physicians who indicate the procedure have to inform about the specific decision aid and other potential evidence-based information as well as the possibility to take the medical records along. For each indication the Institute for Quality and Efficiency in Health Care (IQWiG) develops a decision aid which can be found online. Here again, digital literacy is a prerequisite. In general, decision aids can improve patients’ knowledge about treatment options and reduce their decision conflict compared to usual care. This is demonstrated by a systematic review that included 115 controlled trials with 34,444 participants [36]. However, it is still unclear whether decision aids also have an impact on obtaining an SO.

Interestingly, we found some contradictory results in a previous study among physicians [15]. They stated more frequently that they informed about where to find an SO physician and that the medical records could be taken along. Either this supports the recall bias of the patients or there is also a recall bias among the physicians, which might be based on a social desirability.

With our results we can assume that the SOD is not implemented to the health care system as intended. The items discussed above are mostly neglected by physicians when educating patients. At the same time, patients are interested in obtaining an SO and do so in different ways [31].

We also surveyed parents who made decisions about tonsillectomy/tonsillotomy (and the SO) for their children. This decision might be even more complex and challenging [37]. Unfortunately, our response numbers were too small to draw any conclusions. A study of this particular decision situation would be desirable.

The qualitative results provide insight into how the physician-patient relationship is experienced or affected when the patient is informed about the right to obtain an SO by the physician. We saw that information on SOs does not have a negative influence on the physician-patient relationship; on the contrary, it is perceived as positive and tends to strengthen the trust in the physician-patient relationship. Other studies confirm our findings that the offer of an SO has a positive influence on patients’ trust in the treating physician [38, 39]. Furthermore, the information could also lead to an increase of health literacy because patients deal more reflectively with the disease or the treatment options. As a result, information can certainly lead to patients dealing with the disease in a more autonomous and self-determined way and feeling supported in their decision-making. In summary, information about the right to obtain an SO could lead to an improved relationship of trust and thus to higher patient satisfaction. Thus it could be assumed that the SOD actually reduces the likelihood of SOs insofar as patients are less likely to seek an SO once they have been informed of their right to seek an SO as they perceive the treatment as indicated to be appropriate. Here is a need for further research too.

Despite the effort to reach a structured, independent SO offered to all patients, the SOD is not very well-known among patients. As knowledge about the SOD is largely limited to the information provided by the treating physician so far, there are two options to increase general knowledge of the SOD. Among other things, there seems to be an information deficit on the part of physicians, which in turn leads to an information deficit on the part of patients. On the one hand, one should try to close information gaps on the part of the physicians, and on the other hand, information about an SO should be presented directly to the patient. To raise the knowledge of the physician, one needs to know which barriers (time, organization) are present. We have already learned about physicians’ negative attitude towards the SOD, so it seems that the SOD needs to be adjusted in certain respects to be better accepted. For example the criterion “postdoctoral lecture qualification or advanced trainer for physicians” needed for authorization of SO physicians was heavily criticized as well as the fact that there are not enough SO physicians in rural areas [15]. Since patients with the SOD-assigned indications cannot be identified simply like that, there are again two options. The one is to promote the SOD to the general population through advertisement or general health information. The other is to inform through patient associations or automatically through data entry from insurance companies. In this case, even personalized information can be provided to the potential SO seeker by using patient typologies and an algorithm. However, even the structured SO programs by health insurance marketed in the last years have not resulted in much awareness to date [31]. So advertising for obtaining SOs seems indispensable either way if the general knowledge of it should rise.

### Strength and limitations

To the best of our knowledge, we have performed the first mixed-methods study on patients’ experiences of SOD information. The qualitative interviews allowed an in-depth understanding of patients’ experiences when they were told about the right to obtain an SO and how information is actually provided in reality. As a confirmation, we saw the low numbers of participants in the survey who reported that they were educated about the SOD and provided the related documents. Although we do not know how the education actually took place, we now know at least that it is not perceived by patients as it should be according to the directive.

However, there are certain limitations to our study. The most important limitation is the low response in the quantitative survey. Despite the long recruitment period of almost two and a half years and the incentive, which, however, was only introduced in the last year, only 102 patients could be recruited. Many participating physicians did not recruit any patient. The Covid-19 pandemic drastically reduced the number of elective surgeries and thus the indications were also reduced [40]. At the same time, there was a general decrease in tonsillectomies and hysterectomies in Germany [15, 41]. On the other hand, the long recruitment period and the Covid-19 pandemic may also have biased the results, as patients gave different responses due to structural changes. It could be assumed that there are differences among those sampled at the beginning of the program compared to those who were already in a more advanced stage also because of program adoption. Moreover during the course of the pandemic insecurity in patients may have changed. Regarding the dissemination of the SOD, one could have expected even higher awareness among patients during the course. But the results do not show any differences. Possibly, the pandemic could also have been expected to change behavior with regard to the handling of health information. Again, we did not see this in our results. However, we also suspect that the questionnaire was too long [42]. With the addition of the short questionnaire for shoulder arthroscopy, more subjects were recruited on average. Unfortunately, the short form only asked for information about the directive, but not whether the related documents had been provided. As a second major limitation, we assume a selection bias. Physicians who participated in the study probably had a specific, positive or negative, opinion of the directive. We also assume that they are more aware of the directive as a result of participating in the study, that is, more likely to provide education about the directive or related documents. Information on the SOD can deteriorate in daily routine. The third major limitation is a suspected recall bias. The questionnaire should be completed immediately after indication and the interview should be conducted promptly. When participating in the quantitative survey, we do not know when patients received their indication and when they actually completed the questionnaire. Recollection of education about the directive or even about the patient leaflet may be biased. Participating patients could also be subject to further selection bias. For example, particularly satisfied or particularly dissatisfied patients may have been more likely to participate in the survey. We saw that the health literacy was slightly higher in the participants of the survey than in the general population while professional education was slightly lower [43]. And even with the interviews, it could be that particularly dissatisfied patients wanted to participate to feel validated in their decision. Finally, we would like to point out that possibly an exploratory sequential mixed-methods approach would have led to a deeper understanding of the phenomenon. However, due to recruitment challenges, this study design was not feasible.

## Conclusions

Our results demonstrated that the SOD does not reach the patient as intended. Physicians do not inform sufficiently which might be due to a lack of knowledge or acceptance. Some aspects of the SOD are better communicated to the patient than others. On the other hand we saw that the SOD can support the patients’ decision even though not as intended. Instead, patients which were informed about the SOD feel well cared for by their physician and trust him or her. Further research should explore obtaining an SO according to the directive and the impact of the SOD on patients decision process, as well as tracking patient journeys. In addition, the benefits and impact of patient leaflets on the patient decision-making process should continue to be investigated.

### Electronic supplementary material

Below is the link to the electronic supplementary material.

Supplementary Material 1

Supplementary Material 2

Supplementary Material 3

Supplementary Material 4

Supplementary Material 5

Supplementary Material 6

## Data Availability

All data relevant to the study are included in the article or uploaded as supplementary information. For further questions regarding the reuse of the quantitative data, please contact Barbara Prediger (barbara.prediger@uni-wh.de) and for further questions regarding the reuse of the qualitative data, please contact Susann May (susann.may@mhb-fontane.de).

## Abbreviations

SO: Second opinion
SOD: Second Opinion Directive
HE: Hysterectomy
TE: Tonsillectomy
TT: Tonsillotomy
SA: Shoulder Arthroscopy
