# Supplementary material for: Does the second opinion directive in Germany reach the patient? A parallel-convergent mixed-methods study
Source: BMC Health Serv Res. 2023 Nov 3;23:1198. doi: 10.1186/s12913-023-10197-0 (PMC10623803; doi:10.1186/s12913-023-10197-0)
Supplement: Supplementary file 2 — Supplementary Material 2 [file 12913_2023_10197_MOESM2_ESM.docx]

Supplementary Material File 2

Good Reporting of a Mixed Methods Study (GRAMMS) checklist

| Guideline | Section: page |
| --- | --- |
| Describe the justification for using a mixed methods approach to the research question | Study design: 4 |
| Describe the design in terms of the purpose, priority and sequence of methods | Methods: 4-8 |
| Describe each method in terms of sampling, data collection and analysis | Methods:4-8 |
| Describe where integration has occurred, how it has occurred and who has participated in it | Results - Mixed methods findings: 4-8 |
| Describe any limitation of one method associated with the present of the other method | Strengths and limitations: 18,19 |
| Describe any insights gained from mixing or integrating methods | Results - Mixed methods findings: 14-18 |

O'Cathain A, Murphy E, Nicholl J. The quality of mixed methods studies in health services research. J Health Serv Res Policy. 2008;13: 92-98.
