# Supplementary material for: Does the second opinion directive in Germany reach the patient? A parallel-convergent mixed-methods study
Source: BMC Health Serv Res. 2023 Nov 3;23:1198. doi: 10.1186/s12913-023-10197-0 (PMC10623803; doi:10.1186/s12913-023-10197-0)
Supplement: Supplementary file 3 — Supplementary Material 3 [file 12913_2023_10197_MOESM3_ESM.docx]

Supplementary Material File 3

Interview guide

| Guiding Questions | Check aspects |
| --- | --- |
| How did you experience the treatment from diagnosis to surgery?  What did you feel during this time?  What feelings accompanied you?  How well were you informed about the consequences of the surgery?  How well were you informed about possible alternative treatment options / strategies (incl. Watchful Waiting) and their advantages and disadvantages?  How easy or difficult was it for you to assess the advantages and disadvantages of different treatment options? | Warm up  Experiencing the process of treatment  Process description |
| Did you consider getting a second opinion?  Did you get a second opinion?  If yes:  How did you experience the process? Or how did you go about getting a second opinion?  How did you go about choosing a second opinion?  What was your motivation for seeking a second opinion?  Did the first and second opinions concur?  Did the second opinion help you in reaching your decision?  Would you want to obtain a second opinion again?    If no:  Why did you not seek a second opinion? | Obtaining a second opinion |
| Were you informed of your right to seek a second opinion?  How did you experience the information about the right to seek a second opinion?  If yes:  How did the information take place? And how did you experience it?  What information did you receive from your specialist? Verbally and/ or in writing?  Did you use the decision-making aids?    Did it help you in your decision making? If not, why?  Do you think your doctor's advice that you have a right to obtain a second opinion made you more likely/ or less likely to obtain a second opinion?  What unanswered questions about the process remained?  If no:  Would you have liked your physician to advise you of your right to obtain a second opinion? Why?  Would it have made a difference to you if your doctor had advised you of your right to second opinion? Please explain. | Information about the right to seek a second opinion |
