# Supplementary material for: Does the second opinion directive in Germany reach the patient? A parallel-convergent mixed-methods study
Source: BMC Health Serv Res. 2023 Nov 3;23:1198. doi: 10.1186/s12913-023-10197-0 (PMC10623803; doi:10.1186/s12913-023-10197-0)
Supplement: Supplementary file 4 — Supplementary Material 4 [file 12913_2023_10197_MOESM4_ESM.docx]

Supplementary Material File 4

Patient characteristics in the quantitative study

| Item | Results (%; n/N or Median (IQR)) | | | |
| --- | --- | --- | --- | --- |
|  | Overall | HE | TT/TE | SA |
| According to the guideline, the physician who recommended the surgery has to forward to you some information on the procedure of second opinions. We would like to know which information you received regarding obtaining a second opinion. Please indicate the respective response.  My physician…  …did inform me about the right to a second opinion.  Yes  No  I do not know  No (valid) response  …brought a patient information sheet about the second opinion to my notice.  Yes  No  I do not know  No (valid) response  …made clear to me that I can take away copies of medical records to obtain a second opinion.  Yes  No  I do not know  No (valid) response  …brought to my notice that the second opinion cannot be obtained where the operation is supposed to be conducted (ensuring of independency).  Yes  No  I do not know  No (valid) response  …brought to my notice where I would find information about suitable second opinions.  Yes  No  I do not know  No (valid) response | 73.53%; 75/102  23.53%; 24/102  2.94%; 3/102  0.00%; 0/102  48.04%; 49/102  43.14%; 44/102  7.84%; 8/102  0.98%; 1/102  43.14%; 44/102  44.12%; 45/102  10.78%; 11/102  1.96%; 2/102  41.18%; 42/102  47.06%; 48/102  8.82%; 9/102  2.94%; 3/102  31.37%; 32/102  54.90%; 56/102  11.76%; 12/102  1.96%; 2/102 | 76.19%; 16/21  23.81%; 5/21  0.00%; 0/21  0.00%; 0/21  57.14%; 12/21  33.33%; 7/21  4.76%; 1/21  4.76%; 1/21  33.33%; 7/21  42.86%; 9/21  19.05%; 4/21  4.76%; 1/21  42.86%; 9/21  33.33%; 7/21  19.05%; 4/21  4.76%; 1/21  38.10%; 8/21  42.86%; 9/21  14.29%; 3/21  4.76%; 1/21 | 75.68%; 28/37  21.62%; 8/37  2.70%; 1/37  0.00%; 0/37  35.14%; 13/37  54.05%; 20/37  10.81%; 4/37  0.00%; 0/37  27.03%; 10/37  56.76%; 21/37  16.22%; 6/37  0.00%; 0/37  43.24%; 16/37  45.95%; 17/37  10.81%; 4/37  0.00%; 0/37  24.32%; 9/37  56.76%; 21/37  18.92%; 7/37  0.00%; 0/37 | 70.45%; 31/44  25.00%; 11/44  4.55%; 2/44  0.00%; 0/44  54.55%; 24/44  38.64%; 17/44  6.82%; 3/44  0.00%; 0/44  61.36%; 27/44  34.09%; 15/44  2.27%; 1/44  2.27%; 1/44  38.64%; 17/44  54.55%; 24/44  2.27%; 1/44  4.55%; 2/44  34.09%; 15/44  59.09%; 26/44  4.55%; 2/44  2.27%; 1/44 |
| If you received any of the following text materials please indicate. *Multiple responses are possible*.  Medical records  Information sheet about the second opinion  Consent form  Decision-support tool  No (valid) response | 15.69%; 16/58  13.73%; 14/58  11.76%; 12/58  3.92%; 4/58  27.45%; 28/58 | 38.10%; 8/21  38.10%; 8/21  23.81%; 5/21  4.76%; 1/21  42.86%; 9/21 | 21.62%; 8/37  16.22%; 6/37  18.92%; 7/37  8.11%; 3/37  51.35%; 19/37 | No shoulder! |
| Decision conflict scale  Median  Min  Max  25. Percentile  75. Percentile  Based on x responses | 12.5  0  60.42  4.17  31.25  57 | 8.33  0  56.25  4.17  13.54  20 | 18.75  0  56.25  4.17  13.54  37 | No shoulder |
| Do you wish a second opinion from a physician in your current situation?  Yes  Rather yes  Rather no  No  No (valid) response | 18.63%; 19/102  10.78%; 11/102  27.45%; 28/102  42.16%; 43/102  0.98%; 1/102 | 14.29%; 3/21  14.29%; 3/21  19.05%; 4/21  52.38%; 11/21  0.00%; 0/21 | 5.41%; 2/37  18.92%; 7/37  27.03%; 10/37  48.65%; 18/37  0.00%; 0/37 | 31.82%; 14/44  2.27%; 1/44  31.82%; 14/44  31.82%; 14/44  2.27%; 1/44 |
| What is the cause of your complaints that has led to the recommendation for a surgery? | Specific according to indication | Myome:  57.14%; 12/21  Strong menstruation:  57.14%; 12/21  Endometriosis:  9.52%; 2/21  Uterine prolaps:  0.00%; 0/21  Do not know:  0.00%; 0/21  Other:  52.38%; 11/21  No (valid) response:  4.76%; 1/21 | Recurring tonsilitis:  62.16%; 23/37  Enlarged palatine tonsils:  40.54%; 15/37  I do not know:  2.70%; 1/37  Other:  29.73%; 11/37  No (valid) response:  8.11%; 3/37 | No shoulder |
| How long have the complaints been present already?  Median  Min  Max  25. Percentilel  75. Percentile  Based on x responses | 24  3  360  13.5  60  50 | 24  3  360  10  60  17 | 30  3  360  18  60  33 | No shoulder |
| To what extent are you burdened through your disease in daily life? Try to order your burden on the scale and indicate a value.  1 – Not at all burdened  2  3  4  5  6  7  8  9  10 – Heavily burdened  No (valid) response | 1.72%; 1/58  5.17%; 3/58  3.45%; 2/58  3.45%; 2/58  6.90%; 4/58  17.24%; 10/58  13.79%; 8/58  22.41%; 13/58  8.62%; 5/58  13.79%; 8/58  3.45%; 2/58 | 4.76%; 1/21  4.76%; 1/21  4.76%; 1/21  0.00%; 0/21  9.52%; 2/21  14.29%; 3/21  4.76%; 1/21  14.29%; 3/21  14.29%; 3/21  23.81%; 5/21  4.76%; 1/21 | 0.00%; 0/37  5.41%; 2/37  2.70%; 1/37  5.41%; 2/37  5.41%; 2/37  18.92%; 7/37  18.92%; 7/37  27.03%; 10/37  5.41%; 2/37  8.11%; 3/37  2.70%; 1/37 | No shoulder |
| Tried an alternative treatment method  Yes  No  No (valid) response | Only HE | 42.86%; 9/21  52.38%; 11/21  4.76%; 1/21 | No TT/TE | No shoulder |
| How long do you know the physician who recommended the surgery to you? (Months)  Median  Min  Max  25. Percentile  75. Percentile  Based on x responses | 24  0  240  4.75  84  87 | 72  0  240  30  138  19 | 10  0  168  1  36  30 | 24  0  240  6  81  38 |
| Is the current treatment recommendation a surgical removal or partial removal of the palatine tonsils?  Surgical removal of the palatine tonsils  Surgical partial removal of the palatine tonsils  I do not know  No (valid) response | Only TT/TE | No HE | 45.95%; 17/37  40.54%; 15/37  10.81%; 4/37  2.70%; 1/37 | No shoulder |

| Health literacy (HLS)  Median  Min  Max  25. Percentile  75. Percentile  Based on x responses | 14  4  16  11  15  49 | 14  6  16  12.25  16  18 | 13  4  16  10.5  15  31 | No shoulder |
| --- | --- | --- | --- | --- |
| After the collection of all the information about a disease possible treatment options, some patients prefer that the doctor decides on the treatment. However, others prefer to be involved in the decision. Please go through the following statements and indicate which is the most likely to apply to you. Please only indicate one of the possibilities.  The doctor and I should decide together.  I should decide but strongly consider the physicians opinion.  The physician should decide but should strongly consider my opinion.  The physician should decide by his/herself.  I should decide by myself.  No (valid) response | 58.82%; 60/102  23.53%; 24/102  10.78%; 11/102  1.96%; 2/102  0.00%; 0/102  4.90%; 5/102 | 61.90%; 13/21  28.57%; 6/21  4.76%; 1/21  0.00%; 0/21  0.00%; 0/21  4.76%; 1/21 | 56.76%; 21/37  18.92%; 7/37  16.22%; 6/37  0.00%; 0/37  0.00%; 0/37  8.11%; 3/37 | 59.09%; 26/44  25.00%; 11/44  9.09%; 4/44  4.55%; 2/44  0.00%; 0/44  2.27%; 1/44 |
| Did you have a surgical removal/ partial removal of the palatine tonsils?  Yes  No  No (valid) response | Only parents | Only parents | Only parents 52.63%; 10/19  31.58%; 6/19  15.79%; 3/19 | Only parents |
| Which gender do you have*?  Male  Female  Diverse  No (valid) response | Without shoulder  8.62%; 5/58  84.48%; 49/58  1.72%; 1/58  5.17%; 3/58 | 0.00%; 0/21  100%; 21/21  0.00%; 0/21  0.00%; 0/21 | 13.51%; 5/37  75.68%; 28/37  2.70%; 1/37  8.11%; 3/37 | No shoulder |

| Which gender does your child have?  Male  Female  Diverse  No (valid) response | Only parents | Only parents | Only parents 42.11%; 8/19  47.37%; 9/19  0.00%; 0/19  10.53%; 2/19 | Only parents |
| --- | --- | --- | --- | --- |
| The results of this survey are assessed for different age categories. Therefore, please indicate month and year of your birth.  Median  Min  Max  25. Percentile  75. Percentile  Based on x results | 1976  1938  2001  1961  1985  96 | 1976.5  1949  1989  1972  1980.25  20 | 1989  1962  2001  1983.25  1994  33 | 1961  1938  1998  1955.5  1965.5  43 |
| The results of this survey are assessed for different age categories. Therefore, please indicate month and year of your child’s birth.  Median  Min  Max  25. Percentile  75. Percentile  Based on x results | Only parents | Only parents | Only parents  2014  2004  2018  2011  2016  16 | Only parents |
| Which marital status did you have at the time of the surgery?  Unmarried  Married/Living together in registered civil partnership (same-gender)  Divorced/Annulled registered civil partnership (same-gender)  Widowed/Registered civil partner passed away (same-gender)  Married/Living separately in registered civil partnership (same-gender)  No (valid) response | Without SA  46.55%; 27/58  39.66%; 23/58  10.34%; 6/58  0.00%; 0/58  0.00%; 0/58  3.45%; 2/58 | 38.10%; 8/21  33.33%; 7/21  23.81%; 5/21  0.00%; 0/21  0.00%; 0/21  4.76%; 1/21 | 51.35%; 19/37  43.24%; 16/37  2.70%; 1/37  0.00%; 0/37  0.00%; 0/37  2.70%; 1/37 | Single:  15.91%; 7/44  Married:  81.82%; 36/44  No (valid) response:  2.27%; 1/44 |
| Were you living in a partnership with someone in your household at the time of your surgery?  Yes  No  No (valid) response | 68.97%; 40/58  27.59%; 16/58  3.45%; 2/58 | 71,43%; 15/21  23.81%; 5/21  4.76%; 1/21 | 67.57%; 25/37  29.73%; 11/37  2.70%; 1/37 | No shoulder |
| What was your highest education status at the time of your surgery?  Abitur/General or subject-specific higher education entrance qualification Realschule leaving certificate (Mittlere Reife) or equivalent degree  Fachhochschulreife, leaving certificate from a Fachoberschule  Hauptschule leaving certificate (Volksschule leaving certificate) or equivalent  Polytechnical Oberschule of the GDR with degree after grade ten  Student attending a full time school for general education  Polytechnical Oberschule of the GDR with the degree after grade eight or nine  Left school without degree  Degree of another type of school  No (valid) response | 43.10%; 25/58  18.97%; 11/58  8.62%; 5/58  8.62%; 5/58  5.17%; 3/58  1.72%; 1/58  0,00%; 0/58  0.00%; 0/58  5.17%; 3/58  8.62%; 5/58 | 23.81%; 5/21  23.81%; 5/21  4.76%; 1/21  9.52%; 2/21  14.29%; 3/21  0.00%; 0/21  0.00%; 0/21  0.00%; 0/21  9.52%; 2/21  14.29%; 3/21 | 54.05%; 20/37  16.22%; 6/37  10.81%; 4/37  8.11%; 3/37  0.00%; 0/37  2.70%; 1/37  0.00%; 0/37  0.00%; 0/37  2.70%; 1/37  5.41%; 2/37 | No shoulder |
| What was your highest professional education status at the time of your surgery?  Vocational qualification (duration at least 1 year) completed  Higher education degree  Without vocational qualification  Vocational qualification ongoing  No (valid) response | 54.90%; 56/102  30.39%; 31/102  4.90%; 5/102  1.96%; 2/102  7.84%; 8/102 | 61.90%; 13/21  19.05%; 4/21  4.76%; 1/21  0.00%; 0/21  14.29%; 3/21 | 43.24%; 16/37  35.14%; 13/37  8.11%; 3/37  5.41%; 2/37  8.11%; 3/37 | 61.36%; 27/44  31.82%; 14/44  2.27%; 1/44  0.00%; 0/44  4.55%; 2/44 |
| For the classification of your place of residence in small town, city of medium size, and big city or metropole we would like to know your postcode at the time of your surgery  High population density  Medium population density  Low population density  No (valid) response | 25.49%; 26/102  36.27%; 37/102  27.45%; 28/102  10.78%; 11/102 | 19.05%; 4/21  23.81%; 5/21  42.86%; 9/21  14.29%; 3/21 | 27.03%; 10/37  32.43%; 12/37  27.03%; 10/37  13.51%; 5/37 | 27.27%; 12/44  45.45%; 20/44  20.45%; 9/44  6.82%; 3/44 |
| How many people did live in your household, including yourself, at the time of your surgery?  Only one person  Multiple persons  No (valid) response  If multiple, how many?  Median  Min  Max  25. Percentile  75. Percentile  Based on x responses | 8.62%; 5/58  87.93%; 51/58  3.45%; 2/58  3  1  7  2  4  47 | 14.29%; 3/21  80.95%; 17/21  4.76%; 1/21  3  1  5  2  4  17 | 5.41%; 2/37  91.89%; 34/37  2.70%; 1/37  3  2  7  2  4  30 | No shoulder |
| How many people were older than 14 years at the time of your surgery?  Median  Min  Max  25. Percentile  75. Percentile  Based on x responses | 2  1  4  2  2  46 | 2  1  4  2  3.5  15 | 2  1  4  2  2  31 | No shoulder |
| For the assessment according to groups (e. g. low income, middle income, high income), we would like to know: How high was the average monthly income after taxes of your household in total at the time of your surgery?  0 – 450 euro  451 – 850 euro  851 – 1000 euro  1001 – 1250 euro  1251 – 1500 euro  1501 – 1750 euro  1751 – 2000 euro  2001 – 2250 euro  2251 – 2500 euro  2501 – 2750 euro  2751 – 3000 euro  3001 – 3250 euro  3251 – 3500 euro  3501 – 3750 euro  3751 – 4000 euro  4001 – 4500 euro  4501 – 5000 euro  5001 – 5500 euro  5501 – 6000 euro  6001 – 7500 euro  7501 – 10.000 euro  10.001 – 20.000 euro  More than 20.000 euro  No (valid) response | 1.72%; 1/58  3.45%; 2/58  0.00%; 0/58  8.62%; 5/58  5.17%; 3/58  0.00%; 0/58  8.62%; 5/58  5.17%; 3/58  3.45%; 2/58  3.45%; 2/58  3.45%; 2/58  5.17%; 3/58  1.72%; 1/58  0.00%; 0/58  8.62%; 5/58  5.17%; 3/58  5.17%; 3/58  3.45%; 2/58  3.45%; 2/58  5.17%; 3/58  0.00%; 0/58  0.00%; 0/58  0.00%; 0/58  18.97%; 11/58 | 0.00%; 0/21  4.76%; 1/21  0.00%; 0/21  19.05%; 4/21  4.76%; 1/21  0.00%; 0/21  9.52%; 2/21  4.76%; 1/21  4.76%; 1/21  4.76%; 1/21  0.00%; 0/21  4.76%; 1/21  0.00%; 0/21  0.00%; 0/21  4.76%; 1/21  9.52%; 2/21  4.76%; 1/21  0.00%; 0/21  0.00%; 0/21  9.52%; 2/21  0.00%; 0/21  0.00%; 0/21  0.00%; 0/21  14.29%; 3/21 | 2.70%; 1/37  2.70%; 1/37  0.00%; 0/37  2.70%; 1/37  5.41%; 2/37  0.00%; 0/37  8.11%; 3/37  5.41%; 2/37  2.70%; 1/37  2.70%; 1/37  5.41%; 2/37  5.41%; 2/37  2.70%; 1/37  0.00%; 0/37  10.81%; 4/37  2.70%; 1/37  5.41%; 2/37  5.41%; 2/37  5.41%; 2/37  2.70%; 1/37  0.00%; 0/37  0.00%; 0/37  0.00%; 0/37  21.62%; 8/37 | No shoulder |
