# Supplementary material for: Does the second opinion directive in Germany reach the patient? A parallel-convergent mixed-methods study
Source: BMC Health Serv Res. 2023 Nov 3;23:1198. doi: 10.1186/s12913-023-10197-0 (PMC10623803; doi:10.1186/s12913-023-10197-0)
Supplement: Supplementary file 5 — Supplementary Material 5 [file 12913_2023_10197_MOESM5_ESM.docx]

Supplementary Material File 5

Patient characteristics in the qualitative study

Patient characterstics of the interviewees

| ID | Sex | Age | Surgery planned or undergone at the time of the interview | Number of inhabitants | Highest qualifications | Second Opinion* |
| --- | --- | --- | --- | --- | --- | --- |
| 101_HE | female | 37 | planned | 100.000 | Masters or Magisters degree/ Diploma | yes |
| 102_TE | female | 30 | undergone | 2500 | Apprenticeship | no |
| 103_HE | female | 65 | planned | 2100 | Apprenticeship | no |
| 104_HE | female | 48 | planned | 2000 | Apprenticeship | no |
| 105_TT | female | 35 | undergone | 6300 | Apprenticeship | no |
| 106_TT | female | 27 | undergone | 24.000 | Apprenticeship | no |
| 107_HE | female | 56 | planned | 12.000 | none | no |
| 108_TE | female | 28 | undergone | 3.600.000 | Masters or Magisters degree/ Diploma | yes |
| 109_TE | female | 35 | undergone | 60.000 | none | no |
| 110_TE | female | 37 | planned | 1.800.000 | Apprenticeship | no |
| 111_HE | female | 45 | undergone | 72.000 | Apprenticeship | no |
| 112_SA | male | 64 | undergone | 30000 | Apprenticeship | yes |
| 113_SA | male | 51 | undergone | 30.000 | Apprenticeship | no |
| 114_SA | female | 56 | planned | 3.600.000 | Masters or Magisters degree/ Diploma | yes |
| 115_TE | female | 20 | undergone | 10.000 | Apprenticeship | yes |
| 116_TT | female | 42 | undergone | 23.000 | Apprenticeship | yes |
| 117_HE | female | 42 | undergone | 300 | Apprenticeship | no |
| 118_SA | male | 22 | planned | 3.600.000 | Apprenticeship | yes |
| 119_SA | female | 71 | undergone | 35.000 | Masters or Magisters degree/ Diploma | no |
| 120_SA | male | 83 | planned | 170 | Apprenticeship | yes |
| 121_TT | female | 40 | undergone | 3800 | Apprenticeship | no |
| 122_SA | female | 60 | undergone | 25.000 | Apprenticeship | yes |
| 123_SA | female | 64 | planned | 180.000 | Apprenticeship | yes |
| 124_SA | female | 75 | undergone | 3.600.000 | Masters or Magisters degree/ Diploma | yes |
| 125_SA | female | 45 | undergone | 20.000 | Apprenticeship | yes |
| 126_SA | male | 81 | planned | 180.000 | Masters or Magisters degree/ Diploma | yes |

*All second opinions were not obtained according to the regulations of the directive
