# Supplementary material for: Does the second opinion directive in Germany reach the patient? A parallel-convergent mixed-methods study
Source: BMC Health Serv Res. 2023 Nov 3;23:1198. doi: 10.1186/s12913-023-10197-0 (PMC10623803; doi:10.1186/s12913-023-10197-0)
Supplement: Supplementary file 6 — Supplementary Material 6 [file 12913_2023_10197_MOESM6_ESM.pptx]

## Slide 1
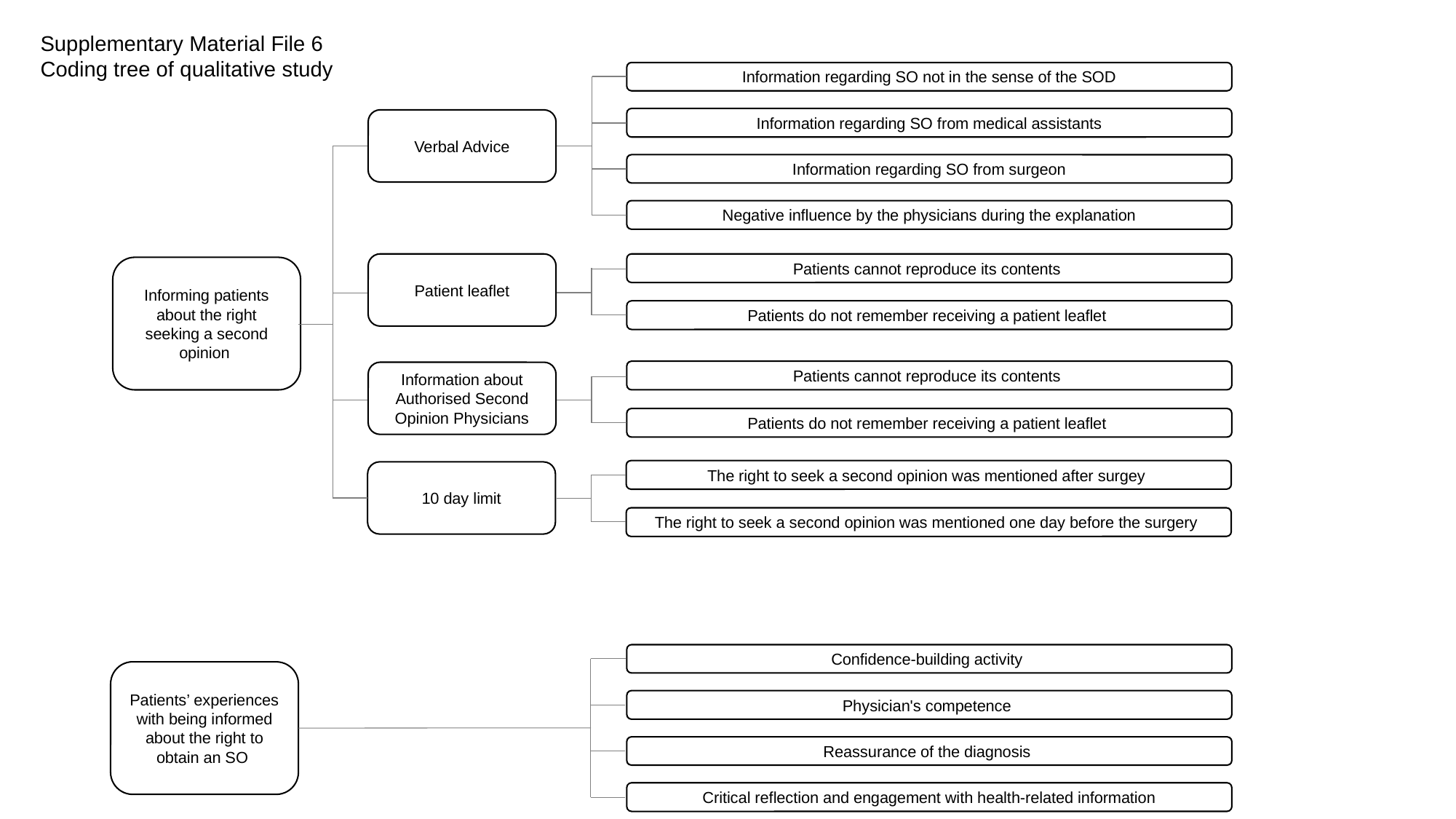

Supplementary Material File 6
Coding tree of qualitative study
Information regarding SO not in the sense of the SOD
Information regarding SO from medical assistants
Verbal Advice
Information regarding SO from surgeon
Negative influence by the physicians during the explanation
Patient leaflet
Patients cannot reproduce its contents
Informing patients about the right seeking a second opinion
Patients do not remember receiving a patient leaflet
Patients cannot reproduce its contents
Information about Authorised Second Opinion Physicians
Patients do not remember receiving a patient leaflet
The right to seek a second opinion was mentioned after surgey
10 day limit
The right to seek a second opinion was mentioned one day before the surgery
Confidence-building activity
Patients’ experiences with being informed about the right to obtain an SO
Physician's competence
Reassurance of the diagnosis
Critical reflection and engagement with health-related information
